# Characterization of defects in mono-like silicon for photovoltaic applications using X-ray Bragg diffraction imaging^1^

**DOI:** 10.1107/S1600576715004926

**Published:** 2015-04-16

**Authors:** M. G. Tsoutsouva, V. A. Oliveira, J. Baruchel, D. Camel, B. Marie, T. A. Lafford

**Affiliations:** a European Synchrotron Radiation Facility, 71 Avenue des Martyrs, Grenoble, F-38043, France; b Université Grenoble Alpes, INES, Le Bourget du Lac, F-73375, France; c Department of Solar Technologies, CEA LITEN, Le Bourget du Lac, F-73375, France

**Keywords:** defect characterization, photovoltaic applications, X-ray Bragg diffraction imaging

## Abstract

Rocking curve imaging (projection and section X-ray topography) has been used to study the generation and propagation of defects at the junctions between and above the seed crystals in mono-like silicon ingots. The images of different kinds of defects such as precipitates, dislocations and twins in the integrated intensity, full width at half-maximum and peak position maps resulting from the experiment have been studied. The qualitative and quantitative information that can be extracted from these maps, in particular the contrast of the images of the various defects, is discussed. These defects have a detrimental effect on solar cell efficiency and their detailed investigation allows clues to be obtained in order to improve the growth process. This work shows that synchrotron X-ray diffraction imaging techniques, because of their high angular resolution (<10^−4^°) and large field of view (several mm^2^), constitute a powerful tool for investigating the initial stages of growth of directionally solidified mono-like silicon.

## Abstract 2

Rocking curve imaging (projection and section X-ray topography) has been used to study different kinds of defects such as precipitates, dislocations and twins in directionally solidified mono-like silicon ingots. The qualitative and quantitative information extracted from the reconstructed integrated intensity, FWHM and peak position maps provides clues about the initial stages of silicon growth.

## 1. Introduction   

Photovoltaic (PV) solar cells directly convert sunlight into electricity. Among the different PV technologies that exist today, crystalline silicon (c-Si) PV is the longest-standing and currently the dominant one with approximately 85–90% of the PV market. The manufacturing process of c-Si modules includes (1) purification of metallurgical silicon to solar-grade poly silicon; (2) melting of poly silicon to form ingots and slicing of these ingots into wafers; (3) transformation of wafers into cells (typically 15 × 15 cm) by creating p–n junctions, metal contacts and back-coating (metallization); and (4) cell assembly, connection and encapsulation into modules.

The Czochralski (Cz) technology is the most common solidification method for producing mono-crystalline silicon (c-Si) ingots for high-efficiency solar cells, but this is an expensive process. Directional solidification is the most widely used technique for producing multi-crystalline silicon (mc-Si) ingots, leading to lower-efficiency solar cells, but at lower cost. The main manufacturing challenge for c-Si solar cells is the growth of high-quality Si crystals able to provide high-efficiency solar cells at a much lower cost. Mono-like silicon is a good candidate for achieving this: it combines the high purity associated with mono-crystalline silicon processing with the lower costs prevalent with multi-crystalline silicon processing. The solidification process is similar to that used for multi-crystalline ingots: directional solidification. A layer of well organized mono-crystalline silicon seeds is employed on the bottom of the crucible. Ideally, silicon grows vertically from the bottom up with the same orientation as the seed layer, thereby creating a large single crystal. However, undesirable defects such as sub-grain boundaries and dislocations can occur at the seed/solidified silicon interface and, dominantly, at the junctions of the seeds, and extend to the whole ingot during the growth process. These defects have a detrimental effect on the solar cell efficiency and their detailed investigation appears important in order to understand the way the initial conditions act on the growth process.

Taking advantage of the high spatial and angular resolution provided by synchrotron light and detection systems, X-ray Bragg diffraction imaging (historically known as ‘X-ray topography’; Lang, 1958 ▶, 1959 ▶), in its fully quantitative version recently developed as rocking curve imaging (RCI) (see below for references), has been used to analyse the generation and propagation of defects originating from the region between the two seeds. The spatial distribution of the lattice distortion can be visualized through a series of X-ray diffraction topographs recorded along the rocking curves, which allows real-space maps of the integrated intensity (INT), full width at half-maximum (FWHM) and peak position (PPOS) to be generated. These maps measure the local crystalline distortion.

In the first part of this work, we investigate the way different kinds of defects such as precipitates, dislocations and twins are manifested in the INT, FWHM and PPOS maps, and we discuss the qualitative and quantitative information that can be extracted from them. In the second part, we apply RCI, in projection and section, to the study of the generation and propagation of defects at the junctions between the seeds in mono-like silicon ingots.

## 2. Experimental procedure   

### 2.1. Transmission rocking curve imaging techniques   

**Figure 1:**
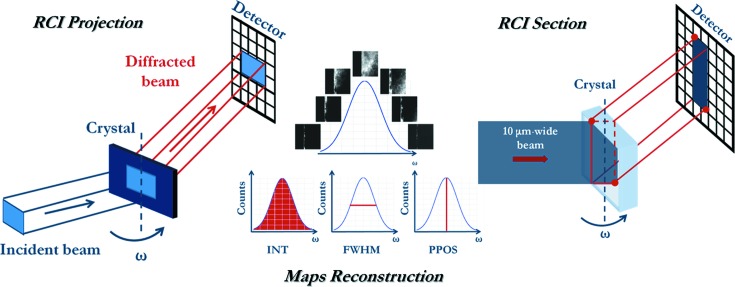
RCI is a quantitative version of monochromatic beam diffraction topography. The crystal is rotated along the diffraction curve for a given set of lattice planes, and the Bragg diffracted beam is recorded on a two-dimensional pixel detector. Each pixel records its own ‘local’ rocking curve, so maps of the whole diffracting area of the sample can be reconstructed. Maps of integrated intensity and FWHM give information about the level of local distortion in each zone of the crystal, and maps of the angular peak position give access to the local departure from the nominal Bragg angle. RCI section topography is based on the same concept except that the incident beam width is reduced by a narrow slit, and thus the diffracted beam corresponds to a virtual slice through the thickness of the sample.

The RCI technique, in both projection (Lübbert *et al.*, 2000 ▶, 2005 ▶; Hoszowska *et al.*, 2001 ▶; Mikulík *et al.*, 2006 ▶; Calamiotou *et al.*, 2007 ▶) and section (Kluender *et al.*, 2011 ▶; Philip *et al.*, 2013 ▶) modes, in transmission, was used to characterize the defects in mono-like Si. Several previous studies (Lafford *et al.*, 2013 ▶; Tsoutsouva *et al.*, 2014 ▶, 2015 ▶; Tran Thi *et al.*, 2015 ▶) have proved that RCI using synchrotron light is a powerful tool for performing this characterization. The principle of these techniques is presented schematically in Fig. 1 ▶.

The experiments were carried out at beamline BM05 at the European Synchrotron Radiation Facility (ESRF). A 20 keV (or 25 keV) beam was extracted from the synchrotron radiation from a bending magnet using a vertically diffracting Si(111) double-crystal monochromator. The sample was positioned to diffract the Si 004 reflection in the horizontal scattering plane (the Bragg angle being 13.19° at 20 keV and 10.52° at 25 keV) or the Si 220 reflection in the vertical scattering plane (the Bragg angle being 9.28° at 20 keV and 7.42° at 25 keV). The diffracted beam profile was recorded using a FReLoN (fast readout low-noise) CCD camera (2048 × 2048 pixels, 16 bit), developed at the ESRF (Labiche *et al.*, 2007 ▶), with three different optics: (i) 0.75 µm pixel size and 1.5 × 1.5 mm field of view, (ii) 1.87 µm pixel size and 3.8 × 3.8 mm field of view, and (iii) 5.8 µm pixel size and 11.9 × 11.9 mm field of view. Given the very low divergence of the synchrotron beam, and the short sample-to-detector distances (35–40 mm), it can be considered as a sensible first approximation, at least for section images, that there is a one-to-one spatial correspondence between one pixel of the camera and a particular ‘voxel’ in the crystal. This voxel corresponds to the intersection between the sample volume illuminated by the beam and a geometrical tube obtained by back tracing the pixel of the detector along the diffracted direction. This simple definition appears to be valid for a range of crystal perfections, which include the ones we are concerned with in the present work, neither ‘perfect’ nor ‘too imperfect’, as will be discussed in more detail at the end of §3.1.

The spatial resolution of the image of the volume investigated in an RCI projection is defined by the pixel size on a plane parallel to the detector plane, but can be much larger in the direction of the diffracted beam, because the signal is integrated over the whole thickness of the sample. The series of rocking curve images was recorded during sample rotation (ω scan) with angular steps of 0.0002° over a range of ∼0.1° across the Bragg angle. Each pixel in the stack of images therefore contains a local rocking curve. From the stack, maps of the INT, the FWHM and the PPOS were extracted to give information about the crystal perfection, the level of distortion and the rotation of the crystal lattice.

RCI section maps show the images of the distortions caused by defects within, to a very good approximation for slightly distorted crystals, a virtual slice defined by the intersection of the crystal and the 10 µm-wide beam. The beam is incident on the sample through a multi-slit mask consisting of an absorbing gold layer deposited on a silicon wafer, which allows the transmission of parallel micro-beams 10 µm wide spaced 0.5 mm apart. One side of the section image corresponds to the entrance surface for the X-rays and the other side to the exit surface (Fig. 1 ▶). This complements the projection technique where, on the image, signals are integrated through the thickness of the sample.

### 2.2. Integrated intensity, full width at half-maximum and peak position maps   

The three maps (INT, FWHM and PPOS) we extract routinely from the RCI data (*i.e.* the intensities recorded in the various pixels of the detector as a function of the sample angular position) provide complementary information. The INT map gives features very similar to those obtained by the well established Lang topography (Lang, 1958 ▶, 1959 ▶) and synchrotron radiation white beam topography techniques. The FWHM map is a quantitative measurement of the local distortion and the general evolution of its variations over the sample. Under low-absorption conditions, which correspond to our case, the FWHM map should produce results qualitatively very similar to those of the INT map, and thus higher intensity and FWHM values correspond to an increased gradient of distortion of the diffracting volume.

The PPOS map provides information on the local departure from the nominal Bragg angle. This departure includes contributions mainly from lattice rotations (tilts) but also from lattice plane spacing variations (strain). This information was obtained, in the past, in a less compact and quantitative way, by the direct observation of the Bragg diffracted recorded images. In white beam techniques, many rotations can be identified, and their value estimated, through the analysis of the image shape (‘orientation contrast’). This has been used to distinguish sub-grains, domains or different phases [many examples are given by, for instance, Weissmann *et al.* (1984 ▶)] and more recently, by simulating these image shapes, to investigate a mechanism of deformation of ice (Philip *et al.*, 2015 ▶). In monochromatic beam techniques (‘plane-wave topography’) this departure from the nominal Bragg angle can be retrieved by the observation of various images recorded at different angular positions of the rocking curve or, for more deformed crystals, by combining these images to produce ‘zebra patterns’ (Ishikawa *et al.*, 1986 ▶; Bowen & Tanner, 1998 ▶). This monochromatic approach allowed the investigation of long-range strain, and/or the determination of the variation of tilts and lattice parameters in different zones of the crystal, for instance neighbouring growth sectors. Again many examples are given by Weissmann *et al.* (1984 ▶). Let us finally note that a much used, and very useful, ‘rocking-curve-analysis-based’ approach is reciprocal space mapping (Bowen & Tanner, 1998 ▶; Holy *et al.*, 1999 ▶), which allows the tilt and strain contributions (coupled in our results) to be distinguished. (Ways to disentangle both contributions in RCI exist but have not been applied in the present work.)

By combining the INT, FWHM and PPOS maps, we obtain extra information with respect to that provided by classical topography techniques, especially in the case of distorted crystals. The PPOS maps, less usual, are those mainly discussed in the present paper.

## 3. Results and discussion   

### 3.1. Observation of defects on RCI maps   

It is well known that the contrast of defects in X-ray Bragg diffraction imaging (‘topography’) arises from the properties of the incident beam, the specimen and the detector. As indicated above, RCI constitutes a quantitative version of the X-ray Bragg diffraction techniques, and all the work that has been performed to explain the contrast of the defects observed on these X-ray images [see for instance the overview by Tanner in the book edited by Authier *et al.* (1996 ▶)] is also valid in our case.

A defect shows up in the diffraction images through its associated ‘effective misorientation’, 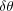, which corresponds to the departure from the Bragg angle, θ_B_, associated with the presence of the defect. 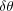 is written, in direct space (Authier, 2001 ▶), as
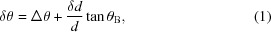
where 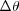 is the component of the local rotation of the lattice planes about the normal to the diffraction plane (containing the incident and diffracted wavevectors, 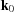 and 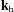, respectively) and 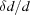 is the local relative variation of the lattice parameter. Expressed in terms of the reciprocal lattice vector, the effective misorientation is 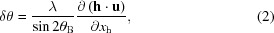
where λ is the wavelength of the diffracted beam, **h** is the diffraction vector, **u** is the displacement vector and 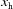 is a coordinate in the direction of the diffracted beam. This relation shows clearly that atomic displacements in the Bragg plane, such that 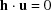, have no influence on 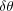, the defect not being visible in this Bragg spot.

A predominant contrast mechanism, in the low-absorption and integrated intensity case, is the ‘direct’ (or ‘kinematical’) image, where the defect image is characterized by enhanced intensity with respect to the perfect crystal matrix (Tanner, 1976 ▶; Klapper, 1991 ▶; Authier *et al.*, 1996 ▶). This image originates from an area surrounding the defect where the effective misorientation, 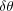, is higher than the intrinsic diffraction width, 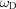. Images corresponding to this mechanism are observed on the INT maps we extract from the RCI data. This also applies to the FWHM maps, the area producing the direct image displaying a larger distortion than the matrix. The FWHM in a given pixel reflects the distribution of the effective misorientation δθ within the associated voxel.

As indicated above, the PPOS map shows the departures from the nominal Bragg angle and mainly rests on another very important contrast mechanism, the ‘orientation contrast’. The PPOS maps quantify the misorientations and could help to access the variation of lattice parameter. As said before, a general view of the misorientations within the sample was obtained in the past either by analysing the shape of the image (white beam) or by considering all the images recorded at several angular positions, and/or often by selecting a few of them to produce a zebra pattern (monochromatic beam). The present approach, *i.e.* the determination of a peak position for each of the pixels (or voxels) associated with the sample, provides in practice additional information about the distortion field around defects, which is not usually processed.

An important point to be mentioned is the range of validity of the one-to-one correspondence between the pixel and the voxel, as defined before, within the crystal. This range depends both on instrumental parameters, such as the pixel size of the detector and the sample-to-detector distance, and on the sample perfection: the crystal should be neither ‘perfect’ nor ‘too imperfect’. Let us comment on this last statement. (1) For a ‘perfect’ crystal, such that the diffraction process corresponds to the one described by dynamical theory, and where interference effects are very important, the whole Borrmann triangle, and not only the region crossed by the incident beam, contributes to diffraction. This is not the case in our crystals and experimental conditions (low-absorption case), where no *Pendellösung* fringes are observed on the section topographs and the predominant images are the ‘direct’ ones. (2) A ‘too imperfect’ crystal is such that either (*a*) the strain (δ*d*/*d*) or (*b*) the twist of the lattice planes, α, is big enough to substantially modify the Bragg angle (α being the twist of the lattice planes around the direction defined by the intersection of the lattice planes and the diffraction plane, which contains the incident and mean diffracted beam vectors). These factors, coupled with a ‘large’ crystal-to-detector distance *L*, can destroy the one-to-one correspondence between the defined voxel and a detector pixel. A general approximation used in this work, where the Bragg angles are small, and therefore where tanθ_B_ ≃ 0.1–0.2, is that the (δ*d*/*d*)tanθ_B_ contribution to the effective misorientation can usually be neglected with respect to the Δθ tilt contribution (except, of course, when Δθ averages out). On the other hand, the twist-associated spatial deviation on the detector, 2*L*αsinθ_B_, remains <1.5 µm in our case, where α < 0.005°, *L* < 40 mm, θ_B_ < 13°. This means that, for pixel sizes in the 1–6 µm range, the approximation of a one-to-one correspondence between the above-defined ‘voxel’ and a detector pixel is sensible (the small departures from these conditions only leading, for the more distorted regions and when using the optics corresponding to the smallest pixel size, to a slightly degraded spatial resolution).

#### 3.1.1. Precipitates   

**Figure 2:**
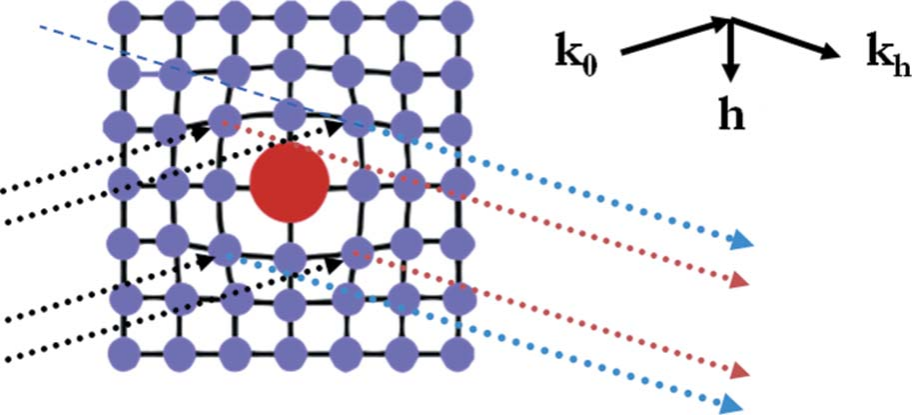
Schematic illustration of a spherical homogeneous precipitate and the distortions it induces in the crystalline matrix, as well as the diffraction conditions that are considered in the discussion of the contrast associated with such a precipitate. The incident beam (dotted black arrows) forms an angle θ_B_ with the diffracting lattice planes. This angle being relatively small (∼9.3°), the image of regions where the rotation of the lattice planes with respect to the matrix orientation is negative (diffracted beam indicated by dotted blue arrows) lies, on the detector, on either side of the image of regions where this rotation is positive (diffracted beam indicated as red dotted lines).

Many precipitates are present in mono-like crystals. Within the Cz-grown seeds, the thermal process can lead the oxygen, already present in the matrix, to form SiO_2_ precipitates (see for instance Bialas & Hesse, 1969 ▶; Lefeld-Sosnowska *et al.*, 1995 ▶; Can Cui *et al.*, 2008 ▶). The SiO_2_ forms at high temperature (973–1073 K), and its thermal dilation is about five times smaller than that of Si. The precipitate therefore exerts compression on the Si lattice, as schematically indicated in Fig. 2 ▶. For our samples, the thermal process associated with the growth led, within the seeds, to a cellular arrangement of dislocations, and no precipitates were observed.

In this work, we will concentrate on the region well above the seeds, in the bulk of the growing crystal. In this region a high density of precipitates, particularly silicon nitride, is occasionally observed. These have appeared in the liquid and have been incorporated into the solid during the growth. Precipitates are also present in the region between the two seeds, but their associated distortion field is difficult to disentangle from that originating from other defects in this rather distorted zone.

The distortion field around a precipitate is a function of many parameters, which include the compositions of the precipitate and matrix, the temperature where the precipitate nucleated, its shape *etc*. The simplest case, which has been widely studied, is that of a spherical homogeneous precipitate (Fig. 2 ▶). On the INT map, its image is well known (Tanner, 1976 ▶; Indenbom & Kaganer, 1985 ▶; Green *et al.*, 1990 ▶): the precipitate shows, in the low-absorption case, two lobes separated by a line of no contrast perpendicular to 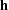, the diffraction vector. This corresponds to the effective misorientation resulting from a strain field
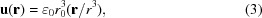
where 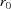 is the radius of the precipitate, and 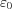 depends on the elastic mismatch between the precipitate and the matrix.

**Figure 3:**
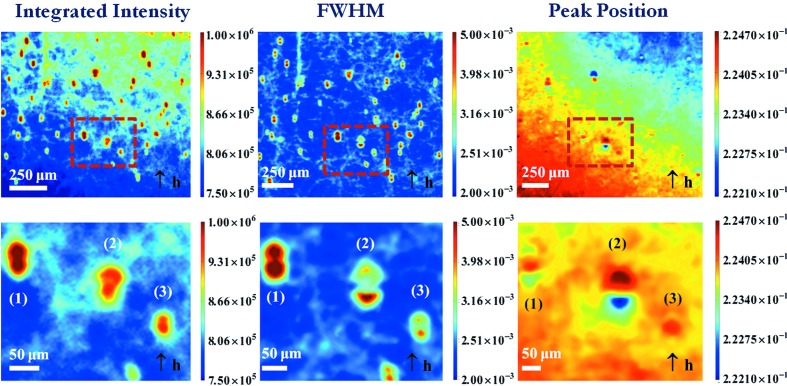
220 diffraction images of a (001) platelet-shaped mono-like Si sample containing precipitates: (left) INT map, (centre) FWHM map, (right) PPOS map. A magnification of the area surrounded by the red rectangle is shown below each of the maps.

Fig. 3 ▶ shows the different 220 maps of an area of a *t* = 0.59 mm-thick platelet-shaped mono-like Si sample, cut perpendicularly to the [001] growth direction at about 40 mm above the bottom of the ingot. The wavelength used was 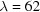
 pm (leading to μ*t* = 0.6, where μ is the linear attenuation coefficient at this wavelength). The distance between the sample and the camera was 40 mm, and the image pixel size was 5.8 µm. Many precipitates are visible on these maps. Images with two lobes are clearly observed on both the INT and FWHM magnified maps.

Let us consider the images expected on the PPOS map under our experimental conditions. These are such that the Bragg angle is ∼9.3°: a simple geometrical consideration shows that a length of ∼35 µm perpendicular to the diffraction vector is projected onto the 5.8 µm pixel of the detector. Let us now consider separately the two components, 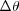 and 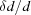, contributing to the effective misorientation, 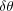. Fig. 2 ▶ shows that, around a spherical precipitate, 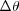 is such that the sample has to be rotated, when moving within the diffraction plane along a direction perpendicular to the diffraction vector, first in one direction in order to satisfy the Bragg condition, then back to the nominal 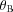 and finally in the other direction. Since the Bragg angle is small, the regions around the precipitate having a positive angular variation of peak position produce an image on the same area of the detector (red dotted arrow in Fig. 2 ▶). The regions where 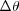 is negative (blue dotted arrows in Fig. 2 ▶) have images lying on either side of the image of the region where 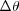 is positive. Let us note that, in the low-absorption case we are concerned with, high integrated intensity and high FWHM are always coupled, indicating that the distorted regions contribute very substantially to the diffracted intensity with respect to the more perfect ones. This is why, while in the blue arrow direction other regions of the crystal also contribute to the diffraction, we can, as a first approximation, concentrate on the contribution corresponding to the more distorted areas, close to the core of the precipitate. The previous arguments therefore indicate that, when having a high enough spatial resolution, the contribution of Δθ to the image of a precipitate on the PPOS map is such that, along the diffraction vector, the peak position appears to be lower, then higher and then lower again than the nominal Bragg angle (*i.e.* that of the matrix). This is what should be observed in the case of a ‘large’ precipitate (distortion field dimension 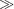
50 µm) and corresponds to what we observe for precipitate (1) of Fig. 3 ▶. If, on the other hand, the precipitate is ‘small’ (distortion field dimension <50 µm) the ‘peak position’ contribution associated with 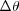 averages out (superposition of the blue and red dotted arrows on the same pixel of the detector) and is not expected to lead to a visible peak displacement. In this case, the only peak displacement that can be observed is therefore that resulting from 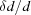, which displays the same sign all around the precipitate (Fig. 2 ▶). This behaviour is observed in Fig. 3 ▶ for the precipitate labelled (3), which exhibits an image in the 40 µm range. The peak position variation observed (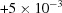
°) corresponds, within the present approximation, to an average 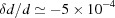 in the neighbourhood of this precipitate. Let us note that the relevant distortion field dimension for a given Bragg diffracted spot is that along the diffraction vector.

But Fig. 3 ▶ shows, in addition, precipitates [such as the one labelled (2)] that, while displaying similar behaviour in the INT and FWHM maps to those labelled (1) and (3), behave very differently in the PPOS map (exhibiting a blue–red contrast). This implies not only that the distortion field extends well beyond the 50 µm indicated above but also that these images do not correspond to the homogeneous/isotropic precipitates assumed. Indeed, many precipitates in mono-like silicon, such as SiC and Si_3_N_4_, do not belong to this simple case.

**Figure 4:**
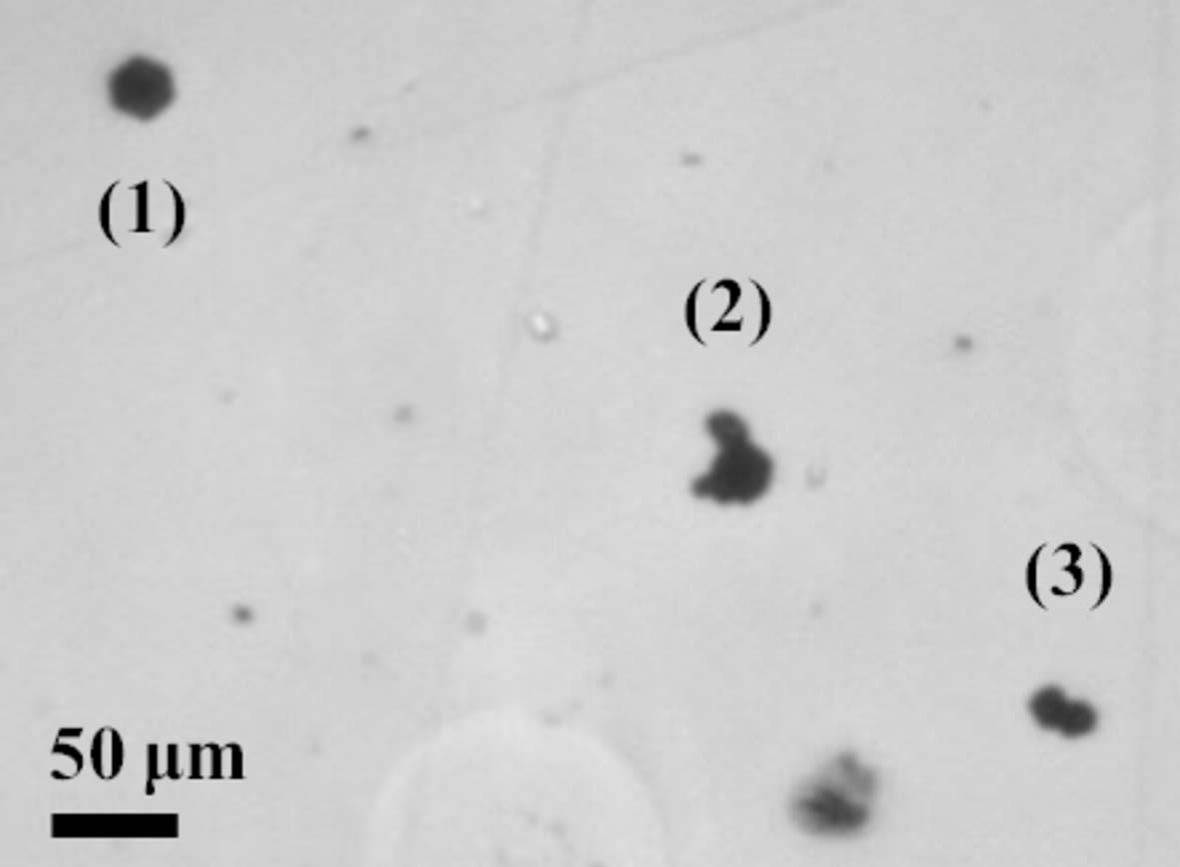
IR image of the area presented in Fig. 3 ▶, which shows that the sizes and shapes of the precipitates differ widely from one another.

Fig. 4 ▶ shows an infrared image of the area presented in Fig. 3 ▶. The dimension of the precipitates, measured along the diffraction vector (Fig. 3 ▶), *i.e.* vertically on Fig. 4 ▶, is such that precipitate (1) appears as ‘large’ (*r*_0_ ≃ 25 µm) and precipitate (3) can be considered as ‘small’ (*r*_0_ ≃ 10 µm), in good qualitative agreement with the RCI results shown in Fig. 3 ▶. The precipitate labelled (2) exhibits an anisotropic shape, which results from the aggregation of several different precipitates, lying at slightly different depths in the sample. Such an anisotropic precipitate can lead to images like those indicated by (2) in Fig. 3 ▶: asymmetry of the lobes in the INT map, different values at the level of the lobes in the FWHM map, and a variation of the peak position when crossing the precipitate along the diffraction vector.

Therefore the present technique allows the observation of the precipitates and provides information about the size and general behaviour of their associated distortion fields.

Let us note that the RCI technique clearly shows, in addition to the precipitates, dislocation images. However, the precipitates observed do not appear to be associated with the generation of dislocations, and therefore they have only a local moderate effect on the distortion of the crystal.

#### 3.1.2. Dislocations   

The width, *D*, of the direct image of a dislocation corresponds to the regions around the dislocation where the effective misorientation, 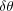, exceeds the intrinsic diffraction width, 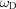, for the perfect crystal. *D* is very close to twice the value of *r*, the distance from the core of the dislocation, where 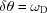. This dislocation width has been checked experimentally by several authors (Miltat & Bowen, 1975 ▶; Klapper, 1991 ▶; Zontone *et al.*, 1996 ▶). For the symmetric Laue case we are concerned with in the present work, 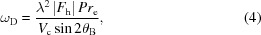
where 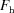 is the structure factor of the reflection used, *P* is the polarization factor (1 or 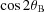, depending on whether the diffraction plane is, in our synchrotron experiment, vertical or horizontal), 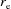 is the classical radius of the electron and 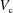 is the volume of the unit cell.

**Figure 5:**
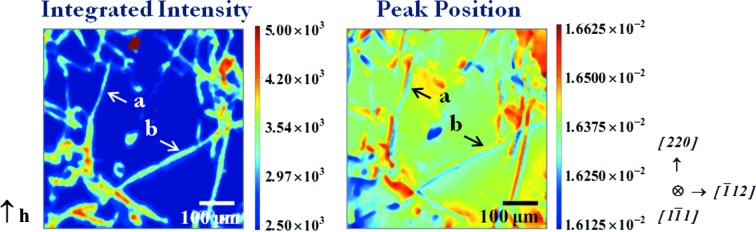
220 INT and PPOS maps of a (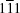) mono-like Si crystal in transmission mode (λ = 50 pm). Sample-to-camera distance 35 mm, 1.87 µm pixel size. The dislocation indicated by a has a dominant screw character, while the one indicated by b has a strong edge component.

Fig. 5 ▶ shows the 220 RCI INT and PPOS maps of an area of a 0.2 mm-thick platelet-shaped mono-like Si sample cut perpendicularly to the growth direction 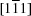. In this case, λ was 50 pm, the sample-to-detector distance 35 mm and the pixel size of the camera 1.87 µm. The images of a series of dislocations are visible. We will consider in particular the dislocation indicated by a, lying close to the [220] direction, which displays a predominantly screw character, and the one indicated by b, lying along [011], which exhibits a strong edge character, as indicated by the analysis of the visibility of their images on a white beam topograph of the same area (not shown).

**Figure 6:**
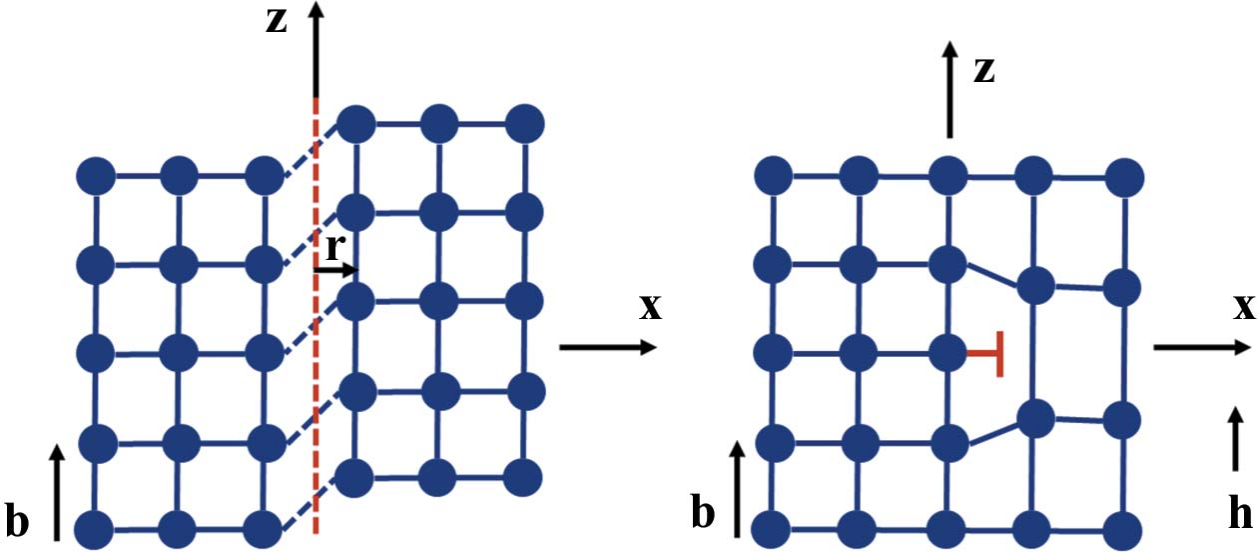
Schematic representations of a screw and an edge dislocation, which aim to facilitate understanding of the text by giving, in a graphic way, the various axes mentioned. The *z* axis is parallel to the diffraction vector and the Burgers vector.

Let us consider (Fig. 6 ▶ *a*) the diffraction from a screw dislocation, with Burgers vector 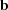, the dislocation line 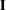 and the diffraction vector 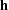, along **z**. The effective misorientation 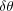 around the dislocation can be deduced from the slope of the helically distorted lattice planes at a distance *r* from the dislocation core. A simple calculation shows that the angle formed by **z** and the perpendicular to the diffracting plane is 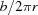 (Klapper, 1991 ▶; Tanner, 1976 ▶). The width of the image of the screw dislocation is therefore, in our case, 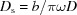, *i.e.* 12 µm (220 reflection, λ = 50 pm). This value is in very good agreement with the average width measured on the INT map for the dislocation indicated by a (Fig. 5 ▶). (The FWHM image, which is not shown in the present paper, is very similar to the INT image.)

The variation of Bragg angle when going, along **y** (Fig. 6 ▶), from one side to the other of the dislocation is
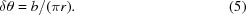



The PPOS map (Fig. 5 ▶) allows measurement of this angle for dislocation a. Let us first note that the width of the dislocation is bigger (∼20 µm) in the PPOS map than in the INT map. This can be qualitatively understood by comparing the way the image is formed in the INT and PPOS cases. In the INT (low-absorption) case and for effective misorientations in the few 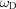 range, the integrated intensity increases, when 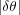 increases, with a zero derivative in the neighbourhood of 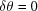 (Ando & Kato, 1970 ▶). This implies that, for small effective misorientations (*i.e.* 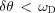), the variation of the integrated intensity with respect to that of the perfect crystal matrix is a second-order phenomenon and therefore that regions with 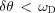 make a negligible contribution to the integrated intensity. But these regions can be visualized on the PPOS map if the angular resolution, 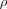, of this map is 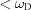. We observe that our experimental procedure leads to 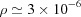
 rad, whereas 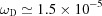
 rad. The fact that the PPOS map is sensitive to smaller effective misorientations implies that more remote regions with respect to the defect core contribute to the PPOS image of the dislocation, which can be, in this way, wider than the INT image.

If we consider that a sensible measure of this variation can be achieved at 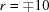
 µm from the core of the dislocation, the calculated value from equation (5) (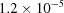
 rad, *i.e.* ∼
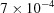
°) is in reasonable agreement with what is observed in the peak position map (∼
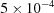
°). Let us point out that equation (5) shows that the variation of the diffraction angle is proportional to the Burgers vector of the dislocation. In the case of a bunch of dislocations, the observed variation of Bragg angle can therefore allow the number of screw dislocations with the same 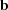 present in the bunch to be estimated. This will be used in the next section.

Let us now consider (Fig. 6 ▶ *b*) an edge dislocation lying along **y**, imaged using diffracting planes such that the diffraction vector 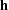 is along **z** (the diffraction plane being *xz*).

It is well known that the width of an edge dislocation 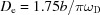 (Klapper, 1991 ▶; Tanner, 1976 ▶). The widths measured for dislocation b in the INT and FWHM maps (FWHM not shown here) are in very good agreement with the calculated width (20 µm).

When moving along **z**, the angle β formed by the trace of the reflecting planes in the *xz* plane and the *x* axis is, at a distance *r* of the core of the dislocation, 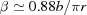. This angle is the main component in the effective misorientation, 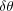 (the 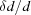 contribution being much reduced by the value of 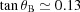). When going from one side to the other of the dislocation line along the *z* axis, β changes its sign when crossing the trace of the extra lattice plane associated with the edge dislocation (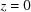), and we have 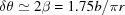. As for the screw dislocation, and for the same reasons, we observe that the width of the image (∼30 µm) in the PPOS map is higher than that observed for the INT map. For 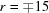
 µm, we have 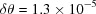
 rad, close to that measured for dislocation b in the PPOS map (∼
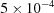
°) (Fig. 5 ▶).

#### 3.1.3. Twins   

Twinning appears in multi-crystalline silicon during crystal growth; thermal grooving, triple junctions (Duffar & Nadri, 2010 ▶) and parasitic grains favour its occurrence. Twins have also been observed in mono-like silicon growing along a 〈111〉 direction (Oliveira *et al.*, 2015 ▶). In the present work, we will restrict the discussion to the contrast these twins, occurring within a single-crystal matrix, produce on the RCI maps.

Twinned regions can show up in Bragg diffraction images because they exhibit ‘structure factor contrast’, *i.e.* the modulus of the structure factor of one region is different from that of the other region, leading to different diffracted intensities, the extreme case being when one of these intensities is zero. For certain sets of diffracting planes, the twinned regions are misoriented. When the twinned regions are not misoriented, the twin boundary may be visible if it is associated with strain concentrations along the boundary (‘dire image’) or, if the crystal is perfect enough, through an effect related to dynamical theory which leads to interference fringes at the boundary (Klapper, 1987 ▶).

**Figure 7:**
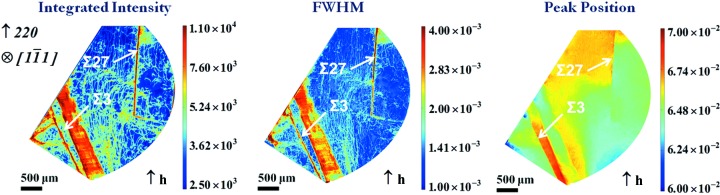
INT, FWHM and PPOS maps, with an indication of the twin boundaries present.

Fig. 7 ▶ shows the 220 RCI maps of an area of a 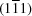 platelet-shaped crystal, cut perpendicularly to the growth direction (λ = 50 pm). The images correspond to a region composed of several twinned domains, which diffract simultaneously for the 220 reflection. These domains are surrounded by other twinned domains which do not satisfy the diffraction condition at this angular position: the straight boundaries (upper and lower left side of the figure) separate the imaged region from these other twinned domains, whereas the rounded boundary (right part of the image) is just the limit of the detection area of our camera.

The twin boundaries were characterized by the electron backscatter diffraction (EBSD) technique in the region imaged and are indicated on the INT map of Fig. 7 ▶. The Σ3 and Σ27 twin boundaries, as well as the lamellae, are observed on both the INT and FWHM maps, indicating that a distortion field is present along these boundaries. In addition, these features display misorientations in the PPOS map, of the order of a few 10^−3^°, confirming that this is ‘imperfect twinning’. A detailed discussion of the formation mechanism of those domains is presented by Oliveira *et al.* (2015 ▶).

### 3.2. Growth defects in mono-like silicon associated with the seed junction   

**Figure 8:**
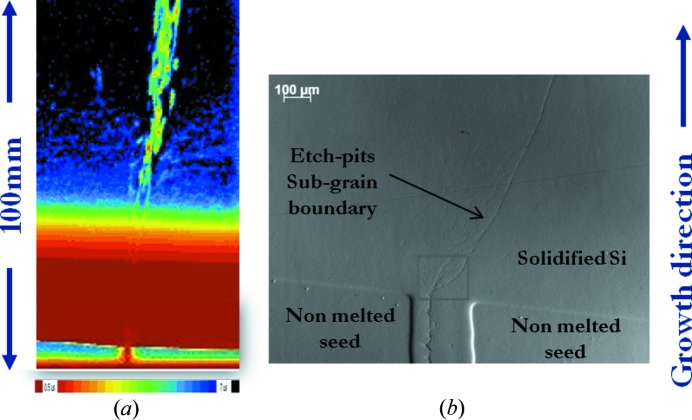
(*a*) Minority carrier lifetime map of a vertical cut across the seed junction of a mono-like silicon ingot and (*b*) optical microscopy image of the solidified area between two seeds on the bottom of the crucible. The sample was mechanically polished on both sides down to a thickness of 0.4 mm and was chemically etched with standard Wright solution for observation of etch pits.

Fig. 8 ▶(*a*) shows the minority carrier lifetime map of a vertical cut across the seed junction of a mono-like silicon ingot. The presence of a sub-grain boundary is observed; this has its origin in the area between the two seeds and leads to the formation of a cascade of dislocations that multiply higher up in the ingot, degrading the minority carrier lifetime. The presence of the sub-grain boundary is confirmed in the optical microscopy image (Fig. 8 ▶ *b*) of the solidified area between two seeds on the bottom of the crucible. Let us note that the direction of the dislocations is very close to the growth direction: in this way, dislocations can adopt directions that are not crystallographically preferred. This fact has already been pointed out in the literature (see for instance Nakajima & Usami, 2009 ▶).

**Figure 9:**
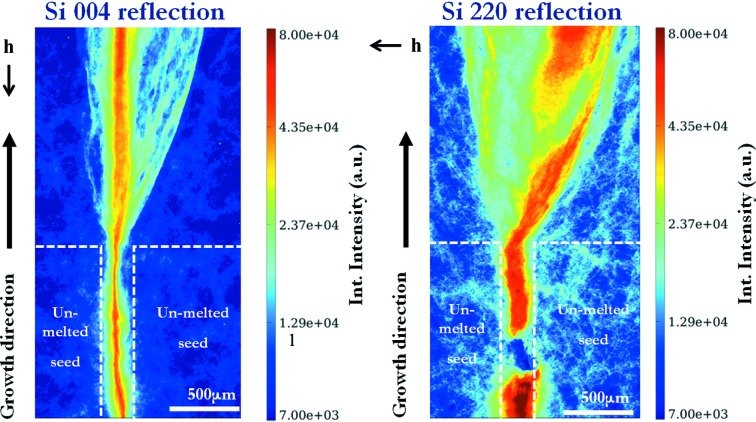
X-ray RCI INT map of the Si 004 and Si 220 reflections from the bottom of the Si ingot at the junction between the two seeds. 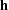 is the projection of the diffraction vector onto the plane of the figure.

In the INT maps of the area near the bottom of the crucible at the junction between the seeds (Fig. 9 ▶) we observe that the solidified silicon has grown epitaxically on the seeds without creating any distorted area at the interface. A highly and inhomogeneously distorted sub-grain boundary-like zone is formed at the junction and develops into a cone-shaped region above the top of the seeds, higher in the ingot along the growth direction. From the INT map of the Si 004 reflection we see that the most highly distorted region is found within the cone; straight lines propagate vertically, following the growth direction. The fact that those lines are not visible in the Si 220 reflection denotes that they display a strong screw component, as will be discussed in more detail later in the present paper. From the INT map of the Si 220 reflection, we also observe a distorted sub-grain boundary that has been generated just above the top edge of the seeds in the junction between them and has propagated towards the right along the growth direction. Its effect in the Si 004 INT map is much less intense and thus it could be said that this sub-grain boundary is composed of dislocations having a strong edge component.

**Figure 10:**
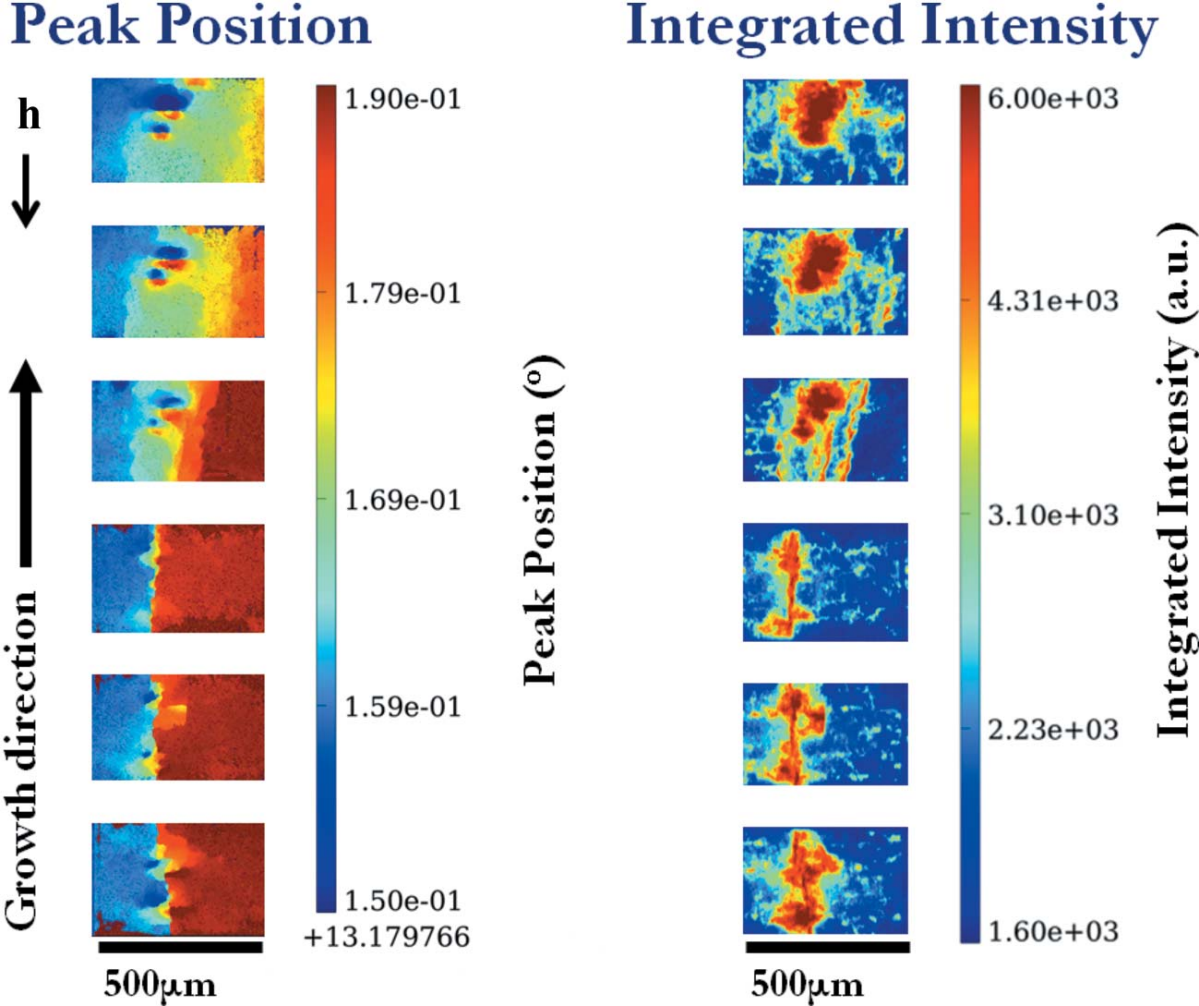
Peak position and integrated intensity section maps of the Si 004 reflection at the junction between the two seeds. 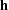 is the projection of the diffraction vector onto the plane of the figure.

RCI section maps give an image of the distribution of distortion through the thickness of the sample. The lower edge of each section corresponds to the beam entrance face of the sample and the upper edge to the exit face. In the present case, RCI section maps of the vertical-cut samples can be considered to be horizontal cuts along the height of the ingot. In the integrated intensity section map of the Si 004 reflection (Fig. 10 ▶) we observe the presence of approximately circular distorted areas in the junction between the two seeds, which increase in size higher up the ingot. This reveals the presence of two bunches of dislocations that pre-existed in the area between the seeds on the bottom of the crucible and grew in conical shapes along the growth direction. These locally distorted regions also create a misorientation of the lattice when going from one side to the other of the bunch of dislocations, affecting the peak position (Fig. 10 ▶). This lattice tilt is associated with the presence of screw dislocations, as explained in §[Sec sec3.1.2]3.1.2. A bunch of screw dislocations behaves like a ‘giant dislocation’ with 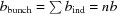 (*b*_ind_ being the individual Burgers vector magnitudes). The misorientation due to the presence of a screw dislocation has been calculated as 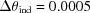
°. The observed relative misorientation is 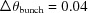
°, and thus we estimate that the bunch contains approximately 80 screw dislocations.

**Figure 11:**
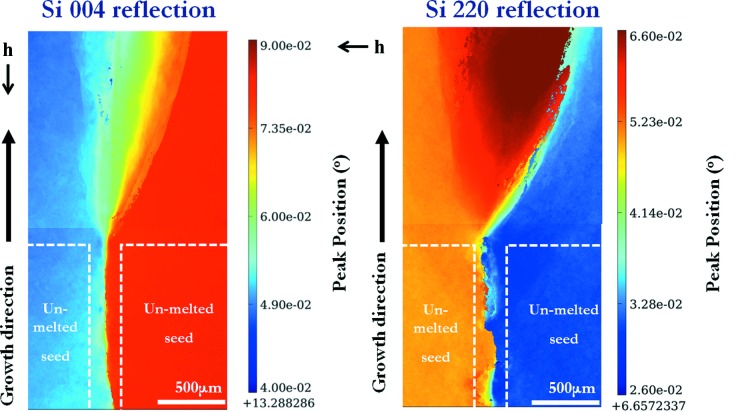
Peak position maps of the Si 004 and 220 reflections at the junction between, and above, the two seeds. 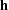 is the projection of the diffraction vector onto the plane of the figure.

From the RCI peak position maps (Fig. 11 ▶), the local lattice misorientations induced by the formation of sub-grain boundaries can be quantified. With the synchrotron X-ray diffraction measurements, even small-angle misorientations with a tilt of a few arcseconds can be distinguished, which is far better than the resolution of EBSD measurements (∼1°).

In the peak position map of the Si 004 reflection (Fig. 11 ▶), above the top of the seeds, a cone-shaped continuous transition zone presenting a gradual variation of the peak position values from the orientation of one of the sub-grains to the orientation of the other is observed.

In contrast, in the Si 220 peak position maps, the transition from one sub-grain to the other is neither monotonic nor continuous. There is an area in which the peak position does not lie between the peaks from the two sub-grains (Fig. 11 ▶). From the same figure, it can be also observed that the misorientation due to the presence of the sub-grain boundary that propagates towards the right along the growth direction rapidly increases with the height. Indeed, it is 0.01° just above the seeds, 0.04° at a position 1.5 mm higher and 0.5° at a position 50 mm above the bottom of the ingot (not shown in the present paper). This process is necessarily associated with a mechanism of rapid multiplication of dislocations. The high dislocation density resulting in the sub-grain boundaries is responsible for the high minority carrier recombination higher in the ingot, revealed by the minority carrier lifetime map (Fig. 8 ▶).

At this point, it is worth mentioning that very low angle sub-grain boundaries that create misorientations as small as 0.01° are recombination-active defects trapping the photogenerated electrons. More information about the role of very small relative misorientations between the seeds in defect generation in mono-like silicon ingots can be found elsewhere (Tsoutsouva *et al.*, 2015 ▶).

## 4. Conclusions   

Rocking curve imaging techniques, in transmission projection and section modes, have been used to investigate the occurrence of defects such as precipitates, dislocations and twins in mono-like silicon samples. The qualitative and quantitative information that can be extracted from the INT, FWHM and PPOS maps has been discussed, and we have pointed out the additional information provided by the PPOS data. A compromise between the level of distortion of the examined sample and the pixel size of the detector should be made, in each investigated case, in order to optimize the experimental procedure and the accuracy of the obtained results. A simple calculation has shown that in the present work the pixel size of the detector and the sample-to-detector distance are well adapted to the level of distortion of the studied samples. These techniques have been applied to a study of the generation and propagation of defects at the junctions between two seeds in mono-like silicon ingots. It was found that the solidified silicon was epitaxically grown on the seeds without creating any distorted area at the interface, but that the unavoidable small relative misorientation between the seeds was responsible for the generation of sub-grain boundaries at the junction. Precipitates were observed, but they do not appear to be associated with dislocations. Dislocations having a strong screw component were found to occur in the area between the seeds and to propagate vertically higher in the ingot along the growth direction. Maps of the diffraction peak position allow the local distortion they induce to be quantified and the number of screw dislocations present in a bunch to be estimated. This work shows that quantitative synchrotron X-ray diffraction imaging, which provides simultaneously a high angular resolution (<10^−4^°) and a field of view of several square millimetres, is a powerful tool for defect observation and characterization and in particular for investigating the initial stages of growth of directionally solidified mono-like silicon.
